# Prevention of Influenza

**Published:** 1892-03

**Authors:** 


### PREVENTION AND TREATMENT OF INFLUENZA.

Attention to the following rules will be found useful for the prevention and cure of influenza: 1. Have pure air by night and day, by the proper ventilation of all rooms. 2. Take frequent long, deep breaths through the nose to clear the lungs. 3. Take sufficient daily exercise in the open air. 4. Take a frequent tepid bath, followed by a rapid toweling, to ensure complete circulation of the blood. An occasional Turkish, or vapor bath, whereby free perspiration is induced, is also beneficial. As to food, the simplest is the best. Fresh fruit, especially oranges and lemons. The juice of the latter, in hot water, taken at bedtime, is very advisable. Farinaceous foods generally; porridges of oatmeal and wheatmeal; eggs, and last, not least, good whole meal bread, comprise a diet fitted for all requirements, provided the following further rules are observed: 1. Thoroughly masticate all food, and eat slowly. 2. Choose that food which best agrees with you; nature is the best judge. 3. Do not eat too much, but leave off feeling you could eat a little more. 4. Avoid hot foods and drinks; these merely create a feeling of warmth, which soon passes off, and the lining also of the throat and stomach is injured thereby, and colds are the more easily caught. 5. Do not take stimulants, such as beer and wines, &c., nor poison your system with drugs.

6. Take tea, coffee or chocolate, in strict moderation, but never drink or eat between meals. 7. Retire to rest early and be up betimes. For those attacked, the following simple hints will probably avail : Sponge throughout with tepid water, and get into bed without drying, taking care to have plenty of clothes over the body. In about half an hour perspiration will follow, when it is best to sponge again with tepid water and again go to bed. This process may be repeated again, when the fever should be expelled through the skin. This remedy is the well known hydropathic cure for ailments of this description, and one found very successful. Should it, however, be considered too heroic a treatment, I would suggest putting the feet in quite hot water (the body being well covered with blankets) until such time as perspiration takes place, when it is advisable to sponge thoroughly with cold or tepid water, and get at once into bed as usual. The food should be of the lightest kind—baked apples, with whole meal bread ; bread and milk, with a lump of butter added. Any of these foods will keep the system well sustained during the twenty-four hours, when the attack should have passed. For drink, orange and lemon-water or barley-water may be freely used. On recovering, gradually return to the diet recommended to those in health.

